# Alterations of Regional Homogeneity in Parkinson’s Disease Patients With Freezing of Gait: A Resting-State fMRI Study

**DOI:** 10.3389/fnagi.2019.00276

**Published:** 2019-10-15

**Authors:** Yanjun Liu, Mengyan Li, Haobo Chen, Xinhua Wei, Guihe Hu, Shaode Yu, Xiuhang Ruan, Jin Zhou, Xiaoping Pan, Ze Li, Zhenhang Luo, Yaoqin Xie

**Affiliations:** ^1^Institute of Biomedical and Health Engineering, Shenzhen Institutes of Advanced Technology, Chinese Academy of Sciences, Shenzhen, China; ^2^Department of Neurology, Guangzhou First People’s Hospital, School of Medicine, South China University of Technology, Guangzhou, China; ^3^Department of Radiology, Guangzhou First People’s Hospital, School of Medicine, South China University of Technology, Guangzhou, China; ^4^Department of Radiation Oncology, Southwestern Medical Center, University of Texas, Dallas, TX, United States; ^5^GYENNO Technologies Co., Ltd., Shenzhen, China

**Keywords:** resting-state fMRI, Parkinson’s disease, freezing of gait, regional homogeneity, movement function

## Abstract

**Objective:**

The purposes of this study are to investigate the regional homogeneity (ReHo) of spontaneous brain activities in Parkinson’s disease (PD) patients with freeze of gait (FOG) and to investigate the neural correlation of movement function through resting-state functional magnetic resonance imaging (RS-fMRI).

**Methods:**

A total of 35 normal controls (NC), 33 PD patients with FOG (FOG+), and 35 PD patients without FOG (FOG−) were enrolled. ReHo was applied to evaluate the regional synchronization of spontaneous brain activities. Analysis of covariance (ANCOVA) was performed on ReHo maps of the three groups, followed by *post hoc* two-sample *t*-tests between every two groups. Moreover, the ReHo signals of FOG+ and FOG− were extracted across the whole brain and correlated with movement scores (FOGQ, FOG questionnaire; GFQ, gait and falls questionnaire).

**Results:**

Significant ReHo differences were observed in the left cerebrum. Compared to NC subjects, the ReHo of PD subjects was increased in the left angular gyrus (AG) and decreased in the left rolandic operculum/postcentral gyrus (Rol/PostC), left inferior opercular-frontal cortex, left middle occipital gyrus, and supramarginal gyrus (SMG). Compared to that of FOG−, the ReHo of FOG+ was increased in the left caudate and decreased in the left Rol/PostC. Within the significant regions, the ReHo of FOG+ was negatively correlated with FOGQ in the left SMG/PostC (*r* = −0.39, *p* < 0.05). Negative correlations were also observed between ReHo and GFQ/FOGQ (*r* = −0.36/−0.38, *p* < 0.05) in the left superior temporal gyrus (STG) of the whole brain analysis based on AAL templates.

**Conclusion:**

The ReHo analysis suggested that the regional signal synchronization of brain activities in FOG+ subjects was most active in the left caudate and most hypoactive in the left Rol/PostC. It also indicated that ReHo in the left caudate and left Rol/PostC was critical for discriminating the three groups. The correlation between ReHo and movement scores (GFQ/FOGQ) in the STG has the potential to differentiate FOG+ from FOG−. This study provided new insight into the understanding of PD with and without FOG.

## Introduction

Parkinson’s disease (PD) is a kind of neurodegenerative disease characterized by motor deficits (Villarreal et al., 2018). Freezing of gait (FOG) is a disabling symptom characterized by brief episodes of an inability to take a step or taking extremely short steps that typically occurs on initiating gait or on turning while walking (Nutt et al., 2011). Although FOG is more commonly observed in PD patients with advanced disease stages and old age, it may also occur in the early stage of idiopathic PD. Nearly 50% of PD patients suffer from FOG (Macht et al., 2007). Though FOG is transient and lasts for only a few seconds, it greatly impacts the quality of life of affected patients.

In the most recent decade, an increasing number of neuroimaging studies have focused on exploring the pathophysiology of PD patients with FOG by using different imaging modalities (Bartels and Leenders, 2008; Fasano et al., 2015) such as positron emission tomography (PET) (Park et al., 2009; Bohnen et al., 2014), functional near-infrared spectroscopy (fNIRS) (Maidan et al., 2015), diffusion tensor imaging (DTI) (Schweder et al., 2010; Herman et al., 2013), and functional magnetic resonance imaging (fMRI) (Tessitore et al., 2012b; Wang et al., 2016; Li et al., 2018). FOG in PD patients is suggested to be associated with abnormalities in motor, executive, cognitive, and affective functions (Amboni et al., 2008; Shine et al., 2013b). It has been synthesized that freezing occurs through a neural pathway in which the transient increase in inhibitory basal ganglia output leads to hypoactivity within the gait-coordinated brainstem, which may be caused by dopaminergic depletion in the striatum and hyperactivity in the subthalamic nucleus (Lewis and Shine, 2016). However, the pathophysiological mechanisms of FOG are not yet fully understood.

Resting-state fMRI (RS-fMRI) reflects alterations in spontaneous brain activities by measuring blood-oxygen-level-dependent (BOLD) signals (Fox and Raichle, 2007), and regional homogeneity (ReHo) evaluates signal synchronization by calculating the concordance of temporal change in BOLD signals within local brain regions (Zang et al., 2004). ReHo has been used to evaluate the differences between PD patients and normal controls (NC) (Li et al., 2016; Pan et al., 2017), and significant differences have been observed in the motor- and executive-related brain regions of PD patients, including the prefrontal cortex (Choe et al., 2013; Borroni et al., 2015), inferior parietal lobule (Wu et al., 2009), basal ganglia (Wang et al., 2018), precentral gyrus (Li et al., 2016; Wang et al., 2016), and cerebellum (Jiang et al., 2016). PD patients with gait disturbance are observed to have impaired coordination of movement and locomotion (Plotnik et al., 2008; Peterson et al., 2012). Therefore, it is hypothesized that PD with FOG (FOG+) may exhibit altered local signal synchronizations of neural activities in comparison to PD without FOG (FOG−) and NC. In this study, ReHo was employed to investigate the regional synchronizations of spontaneous brain activities in FOG+. Moreover, due to the altered movement performance in PD, the neural correlations of movement function in clinical assessment were also explored within the whole brain for both FOG+ and FOG−.

## Materials and Methods

### Subjects and Clinical Assessments

In this study, 37 NC and 72 subjects with a diagnosis of PD were investigated. The NC were healthy subjects with no history of neurological disease, no symptoms of PD, and no disorder of cognitive function. PD patients were diagnosed according to the clinical criteria of the Movement Disorder Society (Postuma et al., 2015). The exclusion criteria for PD patients: severe comorbidity disease (cardiovascular disease, respiratory disease, and malignant tumor, etc.), a history of surgical operations (thalamotomy and posteroventral pallidotomy, deep brain stimulation (DBS), and organ transplantation, etc.), or a pacemaker/metal implanted in their body, which is forbidden in MRI scanning. Among the 72 PD patients, 35 patients were included as FOG+ according to two criteria: (1) rating scores >0 for the third item in the freezing of gait questionnaire (FOGQ), which was described by Giladi et al. (2000) as “Do you feel that your feet get glued to the floor while walking, making a turn or when trying to initiate walking (freezing)?”; (2) based on the former criteria, OFF-FOG patients whose symptoms of FOG were improved after drug therapy were included. The other 37 patients were grouped as PD without freezing of gait (FOG−). In addition, FOGQ was employed to evaluate the severity of FOG performance (Giladi et al., 2000). Other clinical assessments were also made across all subjects with PD. Ratings on the Hoehn and Yahr system (H&Y) (Hoehn and Yahr, 1998) were collected to evaluate the severity of PD symptoms. The gait and falls questionnaire (GFQ) was applied to evaluate the gait and falls risk (Giladi et al., 2000). The motor part of the Unified Parkinson’s Disease Rating Scale (UPDRS-III) was also applied. For the motor assessments of PD patients, both FOGQ and GFQ assessments were made for the most severe OFF medication state based on their experience over the last week, and the UPDRS-III rating was assessed for the ON medication state. Non-motor symptoms of cognitive function were evaluated by montreal cognitive assessment (MOCA) (Nasreddine et al., 2005) and mini-mental state examination (MMSE) (Folstein et al., 1975). The levodopa equivalent daily dose (LEDD) of all of the PD patients was also collected. All subjects were recruited by the Guangzhou First People’s Hospital from May 2017 to September 2018.

### Data Acquisition

All subjects (37 NC, 37 FOG−, and 35 FOG+) were enrolled in 3.0T SIEMENS MRI scanning and were required to lie quietly in the scanner, staying awake with eyes closed. All of the PD patients were in the ON medication state when the MRI scanning was performed. Both functional and structural MRI images were obtained. The RS-fMRI was obtained by echo-planar imaging (EPI) with the following parameters: repetition time (TR) = 2000 ms; echo time (TE) = 21 ms; slice thickness/gap = 4 mm/0.6 mm; acquisition matrix = 64 × 64; flip angle = 78°; in-plane resolution = 3.5 mm × 3.5 mm; FOV = 224 × 224 mm^2^. Sagittal T1-weighted images were obtained with the following parameters: TR/TE = 1900 ms/2.22 ms; acquisition matrix = 256 × 215; flip angle = 9°; in-plane resolution = 0.488 mm × 0.488 mm; slice thickness/gap = 1 mm/0.5 mm.

### Data Preprocessing

The functional images were preprocessed using the toolkits of DPABI (Yan et al., 2016), the RS-fMRI Data Analysis Toolkit (REST)^1^, and Statistical Parametric Mapping (SPM12)^2^, implemented on a MATLAB platform. Data preprocessing included removal of the first 10 of the 220 time points in case of unstable signal quality, slice-timing adjustment (33 slices), head-motion correction, segmentation using a new segment (Ashburner and Friston, 2005) and diffeomorphic anatomical registration through Exponentiated Lie Algebra (DARTEL) (Ashburner, 2007), regression of nuisance covariates (including white matter, cerebrospinal fluid, and Friston’s 24 parameters of head motion) (Friston et al., 1996; Satterthwaite et al., 2013; Yan et al., 2013), spatial normalization to Montreal Neurological Institute (MNI) space by resampling to 3mm × 3mm × 3mm by DARTEL (Ashburner, 2007), a temporal filter with a bandpass of 0.01–0.1 Hz, and removal of linear detrending. Six parameters of head motion (three directions each of rotation and translation) were recorded during the scanning. Subjects with maximal translations exceeding 2.5 mm or rotations over 2.5 degrees were excluded. According to this exclusion criterion, a total of six subjects were excluded from three groups, leaving 35/35/33 subjects for NC/FOG−/FOG+, respectively. Additionally, the mean frame-wise displacement (FD) (Jenkinson et al., 2002) was calculated, which represents the head motion. The mean FD was added as a covariate in the statistical analysis.

### Regional Homogeneity

ReHo evaluates local signal synchronizations by assessing the similarity between the time series of a chosen voxel and those of its neighboring voxels, and Kendall’s coefficient concordance (KCC) is applied to ReHo calculation between a voxel and its 26 neighboring voxels (Zang et al., 2004). KCC-ReHo is a value between 0 and 1. Higher values indicate better local synchronization. Voxel-wise ReHo maps were calculated, and the ReHo maps were then spatially smoothed with a full width at half maximum (FWHM) of 4 mm. Additionally, *Z*-transformation was applied to the ReHo maps for standardization by subtracting the global mean value and then dividing by the global standard deviation. The standardized ReHo Z-maps were applied to the subsequent statistical and correlative analysis.

### Statistical and Correlative Analysis

Analysis of covariance (ANCOVA) was applied to explore the ReHo differences among NC, FOG−, and FOG+, with age, sex, and mean FD Jenkinson as covariates. The resultant *F*-map was corrected by multiple comparisons of the Gaussian Random Field (GRF) with voxel *p* < 0.05 and cluster *p* < 0.05 within a gray matter mask, two-tailed (*F* > 3.83 and cluster size >6750 mm^3^). The surviving voxels were then extracted as a mask in the *post hoc* analysis of ReHo differences between every two groups by two-sample *t*-test with the covariates of age, sex, and mean FD. For the ReHo differences between FOG+ and FOG−, the clinical assessments that demonstrated significant group difference were also controlled as covariates. The resultant *T*-maps were further corrected by GRF with voxel *p* < 0.001 and cluster *p* < 0.05, which is beneficial for avoiding false positives (Woo et al., 2014).

In addition to the whole-brain gray matter, ReHo differences were also investigated within certain brain regions that have frequently been reported in previous motor- and gait-related PD studies, including the basal ganglia (caudate, putamen, and pallidum), sensorimotor cortices, cerebellum, hippocampus, para-hippocampus, and fusiform gyrus (Camicioli et al., 2003; Herman et al., 2014; Pan et al., 2017; Wang et al., 2018; Li et al., 2019). These brain regions were, respectively, extracted from the Automated Anatomical Labeling (AAL) template, which contains 116 brain regions, including 90 cerebrum regions and 26 cerebellum regions (Tzourio-Mazoyer et al., 2002). The corresponding AAL atlas regions were: the bilateral precentral gyrus (PreC, AAL-1,2), postcentral gyrus (PostC, AAL-57,58), supplementary motor area (SMA, AAL-19,20), cerebellum (AAL-91 to 116), caudate (AAL-71,72), putamen (AAL-73,74), pallidum (AAL-75,76), hippocampus (AAL-37,38), para-hippocampus (AAL-39,40), and fusiform gyrus (AAL-55,56). Both ANCOVA and *post hoc* analysis were performed within these regions, respectively, with covariates as in the analysis within whole-brain gray matter. Both ANCOVA and *post hoc* two-sample *t*-test were corrected by GRF with voxel *p* < 0.05 and cluster *p* < 0.05, two-tailed.

Brain regions showing significant differences were extracted as regions of interest (ROIs) for exploring the correlative relationship between signal synchronization and movement function (FOGQ, GFQ). The ReHo value was extracted from ROIs by averaging the values of all voxels within ROI. The Pearson correlation coefficient (statistical significance level *p* < 0.05) was used to quantify the correlation between ReHo and FOGQ/GFQ. Moreover, correlations were also analyzed within AAL templates to examine the neural interactions between regional signal synchronization and movement function across the whole brain.

## Results

### Demographic Characteristics and Clinical Assessments

After exclusion of subjects with excessive head motion, 35 NC, 35 FOG−, and 33 FOG+ remained. There was no significant difference in head motion (mean FD) among the three groups (*p* = 0.2096). Table 1 summarizes the demographic characteristics and clinical assessments of the subjects. A significant difference in age was observed among the three groups (*p* = 0.0001). Specifically, FOG+ showed higher ages than FOG− and NC, while no age difference was found between FOG− and NC (*p* > 0.05). Compared to FOG−, FOG+ had experienced longer disease durations and had higher severity of PD symptoms (H&Y scores) and higher ratings for GFQ and FOGQ. However, FOG+ and FOG− demonstrated no significant differences (*p* > 0.05) on UPDRS-III, MMSE, and MOCA. Note that some PD subjects refused to answer the GFQ and FOGQ and thereby, 35/32 GFQ results remained for FOG−/FOG+ and 34/31 FOGQ results for FOG−/FOG+, respectively.

**TABLE 1:** Demographic characteristics and clinical assessments.

	**NC (*n* = 35)**	**FOG− (*n* = 35)**	**FOG+ (*n* = 33)**	**Statistical *p***
Age (years)	59.57 ± 5.94	62.60 ± 10.22	68.91 ± 8.17	0.0001^a#Δ^
(range)	(47∼81)	(35∼82)	(54∼85)	
Sex (female/male)	24/11	16/19	12/21	0.0232^b^
Education length (years)	11.08 ± 2.84	9.73 ± 3.21	10.64 ± 3.93	0.2416^a^
Disease duration (years)	NA	3.30 ± 3.04	5.81 ± 3.88	0.0077^c^
H&Y scores	NA	2.03 ± 0.52	2.70 ± 0.74	0.000^c^
GFQ (OFF medication)	NA	3.03 ± 2.57	17.41 ± 12.43(*n* = 32)	<0.0001^c^
FOGQ (OFF medication)	NA	1.29 ± 1.32(*n* = 34)	10.55 ± 6.41(*n* = 31)	<0.0001^c^
UPDRS-III (ON medication)	NA	27.48 ± 13.36	31.80 ± 18.35	0.2830^c^
MMSE	27.88 ± 2.10	26.09 ± 3.95	25.39 ± 4.24	0.0153^a*#^
MOCA	25.80 ± 3.13	22.82 ± 5.08	22.29 ± 4.90	0.0034^a*#^
LEDD	NA	319 ± 131	591 ± 387	0.0004^c^
Mean FD (mm)	0.088 ± 0.064	0.076 ± 0.015	0.097 ± 0.069	0.2096^a^

*NC, normal controls; FOG+/FOG−, Parkinson’s disease with/without freezing of gait; H&Y, Hoehn and Yahr; FOGQ, freezing of gait questionnaire; GFQ, gait and falls questionnaire; UPDRS-III, Unified Parkinson’s Disease Rating Scale (part three); MMSE, mini-mental state examination; MOCA, montreal cognitive assessment; LEDD, levodopa equivalent daily dose; FD, framewise displacement; NA, not applicable. Data are given as mean ± standard deviation. ^*a*^Statistical *p*-value by one-way ANOVA test. ^*b*^Statistical *p*-value by chi-square test. ^*c*^Statistical *p*-value by two-sample *t*-test. ^∗^Significant group differences between NC and FOG− indicated by *post hoc* comparisons. ^#^Significant group differences between NC and FOG+ indicated by *post hoc* comparisons. ^Δ^Significant group differences between FOG− and FOG+ indicated by *post hoc* comparisons. Statistical significance level *p* < 0.05 for all tests.*

### Group Differences of Regional Homogeneity

**FIGURE 1:**
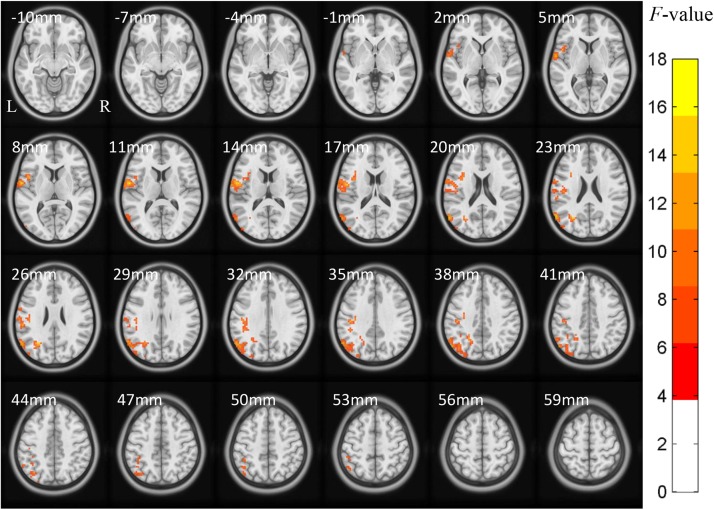
ACONVA analysis of regional homogeneity (ReHo) among NC, FOG–, and FOG+ within gray matter. The *F*-map was corrected by GRF with voxel *p* < 0.05 and cluster *p* < 0.05 (*F* > 3.83, cluster size >6750 mm^3^) within a gray matter mask. L/R, left/right hemisphere; NC, normal controls; FOG+/FOG–, Parkinson’s disease with/without freezing of gait.

**FIGURE 2:**
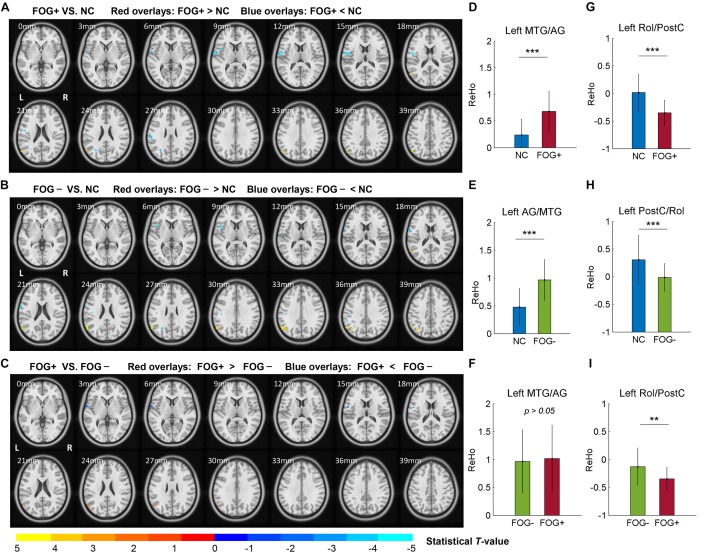
*Post hoc* analysis of ReHo differences among NC, FOG–, and FOG+. **(A)** ReHo differences between FOG+ and NC. **(B)** ReHo differences between FOG– and NC. **(C)** ReHo differences between FOG+ and FOG– (voxel *p* < 0.05, uncorrected). *T*-maps **(A,B)** were corrected by GRF with voxel *p* < 0.001 and cluster *p* < 0.05 (*T* > 3.45, cluster size >135 mm^3^) within the mask of significant brain regions identified by ANCOVA. ReHo signals were extracted from the left MTG/AG **(D–F)** and left Rol/PostC **(G–I)**, which were identified by *post hoc* analysis. L/R, left/right hemisphere; NC, normal controls; FOG+/FOG–, Parkinson’s disease with/without freezing of gait; MTG, middle temporal gyrus; AG, angular gyrus; Rol, rolandic operculum; PostC, postcentral gyrus; ^∗∗^*p* < 0.005; ^∗∗∗^*p* < 0.0005.

The ACONVA analysis demonstrated significant ReHo differences among NC, FOG−, and FOG+. The identification of significant brain regions was based on XjView^3^. It was observed that all significant regions were located in the left cerebrum, including the sensorimotor area of PostC and rolandic operculum (Rol), the posterior middle temporal gyrus (MTG), the parietal gyrus with the angular gyrus (AG) and supramarginal gyrus (SMG), the middle occipital gyrus (MOG), and the opercular part of the inferior frontal gyrus (IFGoper) (Figure 1). Within these significant regions, *post hoc* analysis of two-sample *t*-test was performed between every two groups, corrected by GRF with voxel *p* < 0.001 and cluster *p* < 0.05, two-tailed (*T* > 3.45, cluster size >135 mm^3^). The *T*-map between FOG+ and FOG− was thresholded with an uncorrected *p* < 0.05 (Figure 2C), which failed to survive under GRF correction. The results of between-group ReHo differences are shown in Table 2 and Figure 2. Both FOG+ and FOG− showed higher ReHo than the NC in the left AG, and lower ReHo in the left IFGoper, left MOG, left Rol/PostC, and left SMG/PostC (Table 2 and Figures 2A,B). Moreover, FOG+ achieved higher ReHo than the FOG− in the left MTG and left AG (Table 2 and Figure 2A) and lower ReHo than the FOG− in the left Rol/PostC (Table 2 and Figure 2C).

**TABLE 2:** Brain regions showing significant regional homogeneity (ReHo) differences among NC, FOG−, and FOG+ within gray matter.

**Brain regions**	**BA**	**Cluster size (mm^3^)**	**Peak MNI coordinates (x y z)**	**Peak *T*-value**
**FOG+ vs. NC (Positive/Negative T-value indicates increased/decreased ReHo in FOG+)**				
L-AG	39	567	–54 –69 39	4.32
L-MTG	39	378	–60 –66 21	4.13
L-Rol/PostC	48/22	1269	–60 –3 9	–4.92
L-IFGoper	48	189	–45 6 12	–4.01
L-MOG	19	243	–30 –63 27	–4.14
L-SMG/PostC	48	270	–57 –30 27	–4.15
**FOG− vs. NC (Positive/Negative T-value indicates increased/decreased ReHo in FOG−)**				
L-AG/MTG	39	2268	–60 –66 24	4.83
L-IFGoper	48	297	–48 9 6	–4.71
L-PostC/Rol	43	486	–63 0 18	–4.04
L-MOG	19	162	–27 –63 27	–4.45
L-PostC/SMG	48	270	–45 –21 27	–4.16
**FOG+ vs. FOG− (Positive/Negative T-value indicates increased/decreased ReHo in FOG+)**				
L-MTG/AG	39	405	–54 –66 24	2.70
L-PostC	48	216	–57 0 15	–2.62
L-Rol	48	243	–63 0 3	–2.38

*NC, normal controls; FOG+/FOG−, Parkinson’s disease with/without freezing of gait; L, left hemisphere; BA, Brodmann area; MNI, montreal neurological institute; AG, angular gyrus; MTG/MOG, middle temporal/occipital gyrus; Rol, rolandic operculum; PostC, postcentral gyrus; IFGoper, opercular part of the inferior frontal gyrus; SMG, supramarginal gyrus. *T*-value was the statistical *t* by *post hoc* analysis of two-sample *t*-test.*

**FIGURE 3:**
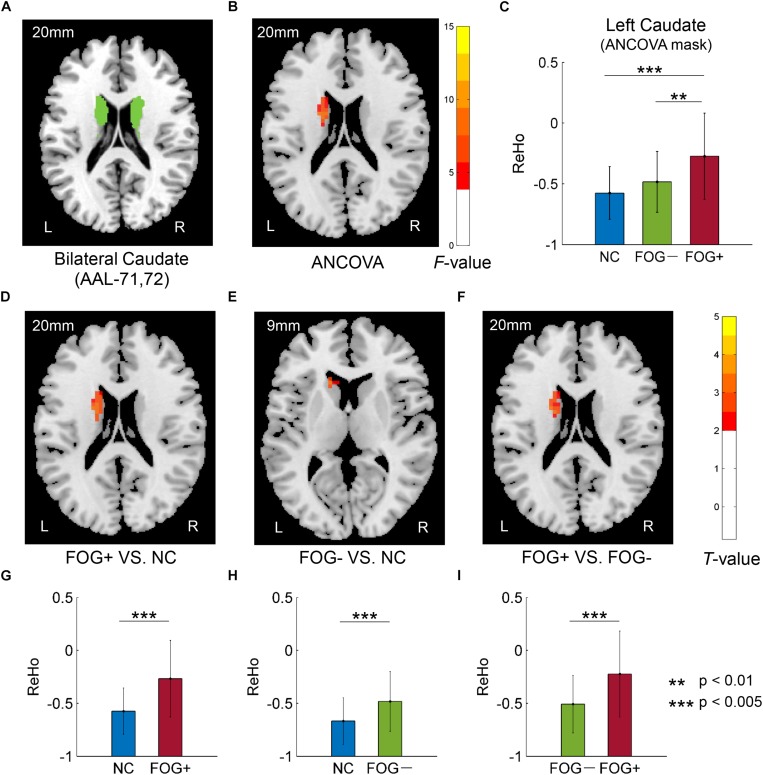
Regional homogeneity differences among NC, FOG–, and FOG+ within the bilateral caudate. **(A)** Bilateral caudate. **(B)** *F*-map of ANCOVA analysis of the three groups, corrected by GRF with voxel *p* < 0.05, threshold of *F* > 3.84 and cluster size >1566 mm^3^. **(C)** FOG+ subjects showed higher ReHo within the left caudate than did FOG– and NC. *Post hoc* two-sample *t*-tests on FOG+ and NC **(D,G)**, FOG– and NC **(E,H)**, and FOG+ and FOG– **(F,I)**. The *T*-map in **(E)** was with a threshold of an uncorrected voxel *p* < 0.05, **(D,F)** were corrected by GRF with voxel *p* < 0.05, threshold of *T* > 2.00 and cluster size >486 mm^3^. L/R, eft/right hemisphere; NC, normal controls; FOG+/FOG–, Parkinson’s disease with/without freezing of gait.

Furthermore, ReHo differences were also examined in the bilateral PreC, PostC, SMA, cerebellum, caudate, putamen, pallidum, hippocampus, para-hippocampus, and fusiform gyrus, respectively. However, significant results were only observed in the caudate (Table 3 and Figure 3). The *F*-map acquired by ANCOVA among the three groups was corrected by GRF with voxel *p* < 0.05 and cluster *p* < 0.05 (threshold of *F* > 3.84 and cluster size >1566 mm^3^) (Figure 3B). ReHo differences were demonstrated between the three groups in the left caudate (Figures 3B,C). *Post hoc* two-sample *t*-testing on the pair groups were performed within the significant left caudate, and the resultant *T*-maps were corrected by GRF with voxel *p* < 0.05 and cluster *p* < 0.05 (threshold of *T* > 2.00 and cluster size >486 mm^3^). FOG+ was observed to have higher ReHo than FOG− (Figures 3F,I) and NC (Figures 3D,G) in the left caudate. While no cluster survived the GRF correction for the *T*-map between FOG− and NC, thresholding with an uncorrected voxel *p* < 0.05 and cluster size of 351 mm^3^ demonstrated higher ReHo in the left caudate in FOG− than in NC (Figures 3E,H).

**TABLE 3:** Brain regions showing significant ReHo differences among NC, FOG−, and FOG+ within the bilateral caudate.

**Groups**	**Multiple comparison correction**	**Cluster size (mm^3^)**	**Peak MNI coordinates (x y z)**	**Peak statistical value**
NC, FOG−, FOG+	GRF *p* < 0.05	1566	–15 0 24	*F* = 15.00
FOG+ vs. NC	GRF *p* < 0.05	1458	–15 0 24	*T* = 5.16
FOG− vs. NC	Uncorrected *p* < 0.05	351	–15 21 9	*T* = 3.44
FOG+ vs. FOG−	GRF *p* < 0.05	918	–18 0 24	*T* = 4.03

*NC, normal controls; FOG+/FOG−, Parkinson’s disease with/without freezing of gait; MNI, montreal neurological institute; GRF, gaussian random field. The *F*-value indicates the statistical value among the three groups according to ANCOVA. The *T*-value indicates the statistical value of the between-group differences according to *post hoc* two-sample *t*-test.*

### Correlative Analysis

**FIGURE 4:**
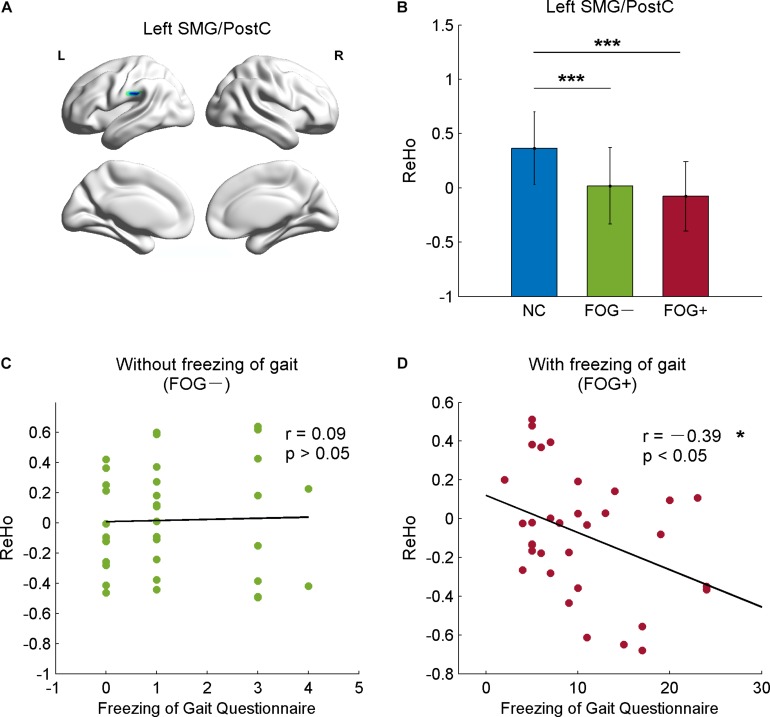
Correlation between ReHo and movement function in the left supramarginal gyrus/postcentral gyrus (SMG/PostC). **(A)** Visualization of left SMG/PostC. **(B)** ReHo of left SMG/PostC. ^∗∗∗^*p* < 0.0001 between FOG+/FOG– and NC, *p* = 0.2408 between FOG+ and FOG–. **(C)** Correlation between ReHo of FOG– and movement function. **(D)** Correlation between ReHo of FOG+ and movement function.

The correlative analysis between local signal synchronization (ReHo) and movement function (GFQ/FOGQ) was performed within significant brain regions identified by *post hoc* two-sample *t*-tests. Among the regions, significant results were observed in the left SMG/PostC (Figure 4A), which was visualized by BrainNet Viewer (Xia et al., 2013). The ReHo values of FOG+ and FOG− were significantly lower than those of NC (*p* < 0.0001), while no difference was found between those of FOG+ and FOG− (*p* = 0.2408) (Figure 4B). The ReHo of FOG+ was negatively correlated with FOGQ in the left SMG/PostC (*r* = −0.39, *p* < 0.05) (Figure 4D), while no significant correlation was found between the ReHo of FOG− and FOGQ (Figure 4C). In addition, neither the ReHo of FOG+ nor the ReHo of FOG− was correlated with GFQ.

**FIGURE 5:**
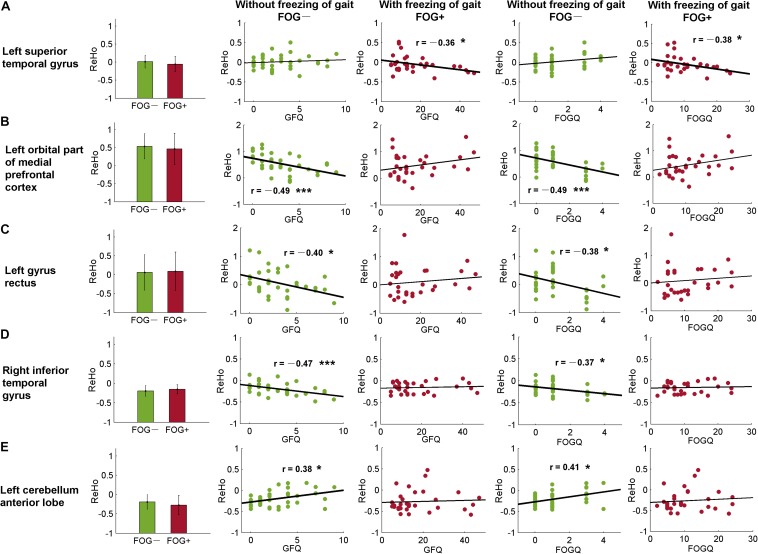
Correlative analysis between ReHo and movement function within automated anatomical labeling (AAL) templates. Significant correlation was found in the **(A)** left superior temporal gyrus, **(B)** left orbital part of the medial prefrontal cortex, **(C)** left gyrus rectus, **(D)** right inferior temporal gyrus, and **(E)** left cerebellum anterior lobe. FOGQ, freezing of gait questionnaire; GFQ, gait and falls questionnaire. Statistical significance notation: ^∗^*p* < 0.05, ^∗∗∗^*p* < 0.005.

As well as from the ROIs identified by the *post hoc* analysis (Tables 2, 3), ReHo signals were also extracted from AAL templates and correlated with GFQ and FOGQ, respectively. Significant results were observed in the left superior temporal gyrus (STG, AAL-81), left orbital part of the medial prefrontal cortex (MPFCorb, AAL-25), left gyrus rectus (AAL-27), right inferior temporal gyrus (ITG, AAL-90), and left cerebellum anterior lobe (CAL, AAL-97) (Figure 5). The ReHo values of FOG+ in the left STG were observed to have negative correlations (*p* < 0.05) with GFQ (*r* = −0.36) and FOGQ (*r* = −0.38) (Figure 5A). The ReHo values of FOG−, meanwhile, were observed to have negative correlations with GFQ/FOGQ in the left MPFCorb (Figure 5B), left gyrus rectus (Figure 5C), and right ITG (Figure 5D) and to be positively correlated with GFQ/FOGQ in the left CAL (Figure 5E). Though significant correlations were observed in the above five brain regions, the ReHo signals extracted from the five sub-templates showed no significant difference (*p* > 0.05) between FOG− and FOG+ according to two-sample *t*-test (Figure 5).

## Discussion

In this study, we assessed ReHo to explore differences in local synchronization among NC, FOG+, and FOG− subjects, and significant results were observed in the left cerebrum. In comparison to NC, FOG+, and FOG− showed increased ReHo in the left AG but decreased ReHo in the left IFGoper, left Rol/PostC, left MOG, and left SMG (Table 2 and Figures 2A,B). Compared to FOG−, the ReHo in FOG+ was decreased in the left Rol/PostC (Figure 2C) and increased in the left caudate (Figure 3). Neural correlation analysis showed that the ReHo of FOG+ was negatively correlated with FOGQ (*r* = −0.39) in the left SMG/PostC (Figure 4) and negatively correlated with GFQ/FOGQ (*r* = −0.36/−0.38) in the STG (Figure 5A).

Previous studies have reported structural changes in FOG+ vs. FOG−, with gray matter volume reductions in the posterior cingulate cortex/precuneus (PCC/PCu) (Tessitore et al., 2012a), left IFG (Pan et al., 2012), inferior parietal lobule (IPL) (Kostić et al., 2012), and AG (Herman et al., 2014). The PCC/PCu, medial prefrontal cortex (MPFC), AG, and posterior MTG are major parts of the default mode network (DMN), where brain activities are active in the resting state but passive in the task-induced state (Raichle et al., 2001). Gray-matter atrophy of the DMN in FOG+ probably leads to dysfunctional brain activities in the resting state. Meta-analysis of the ReHo of PD has been reported to give consistent results in the DMN and motor networks, with increased ReHo in the bilateral IPL, bilateral MPFC, and left cerebellum and decreased ReHo in the right putamen and right PreC (Pan et al., 2017; Wang et al., 2018). Our findings are consistent with the results showing increased ReHo in the DMN, observing increased ReHo in the left MTG/AG (Figures 2A,B,D,E). However, no gait-specificity of ReHo in the DMN was found in PD patients. Under a threshold of an uncorrected voxel *p* < 0.05, FOG+ were observed to have increased ReHo in the left MTG/AG (Figure 2C), while no difference was found between FOG+ and FOG− when the averaged ReHo was extracted from these regions (Figure 2F). A previous study on FOG+ also reported decreased ReHo in the frontal cortex and motor area (Zhou et al., 2018), which suggests that FOG+ subjects have decreased cognitive function and motor function. Our findings also demonstrated decreased ReHo in the IFG and MOG. A resting-state study with connectivity analysis of FOG+ observed decreased connectivity in the frontal and occipital lobes, which correspond to the executive and visual networks (Tessitore et al., 2012b). These findings suggest the disruption of executive and visual functions in FOG+.

One of the most critical findings of this study is that for ReHo in the left Rol/PostC, FOG+ <FOG−<NC, which suggests that this region is most hypoactive in FOG+ (Figures 2G–I). However, when using a threshold of an uncorrected voxel *p* < 0.05 when comparing the ReHo of FOG+ and FOG−, significant difference (*p* < 0.005) was observed in the left Rol/PostC by extracting the averaged ReHo from the surviving voxels (Figure 2I). This suggests that ReHo values in the left Rol/PostC, the sensorimotor areas, are critical features for discriminating the three groups. One interesting finding comes from the fact that all of the significant ROIs among FOG+, FOG−, and NC were located in the left hemisphere of the cerebrum (Figures 1,2 and Table 2). An fMRI study of emotional picture stimuli reported activations of left-brain activities in response to positive pictures and hence suggested that the left hemisphere of the brain is associated with positive emotions (Canli et al., 1998). Altered ReHo in the left hemisphere may reveal decreased positive emotion in FOG+. Previous studies also demonstrate that emotional state affects the motor control of gait (Naugle et al., 2011). Depression and anxiety are also the major emotional symptoms in PD, which exacerbate poor motor performance (Macht et al., 2005; Avanzino et al., 2018). Therefore, cognitive behavioral therapy is recommended for the treatment of patients with FOG (Berardelli et al., 2015).

The basal ganglia, sensorimotor area, cerebellum, hippocampus, parahippocampus, and fusiform gyrus are critical regions involved in the pathophysiology of PD. ReHo differences were also examined within these brain regions. However, significant results were only observed in the left caudate: FOG+ >FOG−>NC (Table 3 and Figure 3). Gray matter atrophy in the left caudate (Jia et al., 2019) and consistently decreased ReHo in the putamen (Pan et al., 2017; Wang et al., 2018) have been observed in PD patients. In our results, meanwhile, PD showed increased ReHo in the left caudate, with FOG+ being the most active. Decreased ReHo may indicate functional deficits caused by diseases, while increased ReHo may be related to a compensatory mechanism for maintaining normal function (Pan et al., 2017). It is suggested that FOG in PD is associated with functional decoupling between the cognitive control network and the basal ganglia (Shine et al., 2013a). Increased ReHo in the left caudate may reflect a compensation of the cognitive control function in PD patients.

Within the significant brain regions, the ReHo of FOG+ was negatively correlated (*r* = −0.39) with FOGQ in the left SMG/PostC (Figure 4). Negative correlation was also observed between ReHo and GFQ/FOGQ (r = −0.36/−0.38) in the STG based on the whole-brain analysis (Figure 5A). A DBS study on the effect of sub-thalamic nucleus stimulation in PD reported a positive correlation between motor scores and metabolic activity in parietal-temporal sensory-related brain areas (Chul et al., 2007). Activation of brain activity was found in the temporal lobe during a memory paradigm fMRI study after rehabilitation (Díez-Cirarda et al., 2017). These findings indicate that brain activities in the motor area and temporal lobe are associated with an improvement in motor performance.

There are two limitations to this study. The demographic characteristics of age and sex are not properly matched among FOG+, FOG−, and NC. The age of FOG+ is significantly higher than that of FOG− and NC. PD patients with advanced age and a higher stage of disease progression are more likely to experience FOG (Zhang et al., 2016). In this study, age and sex were included as covariates in the statistical analysis to regress out the unmatched confounds. The second limitation comes from the insufficient sample size. In our future work, more subjects will be enrolled for a better understanding of the neuroimaging features of PD patients with freezing of gait.

## Conclusion

ReHo was used to explore the regional signal synchronization of brain activities in PD patients. The results suggest that the brain activities of PD patients with FOG were the most active in the left caudate and the most hypoactive in the left Rol/PostC. The correlation analysis between ReHo and movement scores (GFQ/FOGQ) in the left STG provides the potential to stratify PD patients with and without FOG. This study provides new insight for understanding PD patients.

## Data Availability Statement

The datasets generated for this study are available on request to the corresponding author.

## Ethics Statement

This study was approved by the Institutional Review Board (IRB) of Guangzhou First People’s Hospital. Written informed consent was obtained from all subjects.

## Author Contributions

YL wrote the manuscript. YL, ML, and XW conceived of the idea and performed the literature review. YL, ML, XW, HC, GH, and SY performed the data analysis. XW, XR, ZLuo, and JZ contributed to the data collection. All authors interpreted the results, reviewed the manuscript and joined the discussion of the manuscript.

## Conflict of Interest

ZLuo was employed by the company of GYENNO Technologies Co., Ltd. The remaining authors declare that the research was conducted in the absence of any commercial or financial relationships that could be construed as a potential conflict of interest.

## Footnotes

1 http://www.restfmri.net/forum/REST

2 http://www.fil.ion.ucl.ac.uk/spm/

3 http://www.alivelearn.net/xjview/
